# Crowd navigation in a multi-room environment: a model predictive control framework for mobile robots

**DOI:** 10.3389/frobt.2026.1812386

**Published:** 2026-07-27

**Authors:** Giovanbattista Gravina, Francesco D’Orazio, Michele Cipriano, Tommaso Belvedere, Giuseppe Oriolo

**Affiliations:** 1 Humanoids and Human Centered Mechatronics (HHCM), Istituto Italiano di Tecnologia, Genova, Italy; 2 Department of Computer, Control and Management Engineering, Sapienza University of Rome, Rome, Italy; 3 CNRS, Université de Rennes, Inria, IRISA, Rennes, France

**Keywords:** active perception, control barrier functions, crowd navigation, integrated planning and control, model predictive control, sensor fusion, sensor-based control

## Abstract

Mobile robots operating in human-populated environments must navigate complex, multi-room spaces while ensuring safety, i.e., generating collision-free motion. In this study, we present a sensor-based model predictive control (MPC) scheme designed for safe crowd navigation in such non-convex environments. The proposed framework decomposes the free space into a set of overlapping convex regions to construct a topological graph, enabling a high-level planner to compute optimal sequences of traversable areas. To effectively perceive the crowd, the system employs a robust perception pipeline that fuses 2D LiDAR data with semantic information from an RGB-D camera, utilizing Kalman filters (KFs) to estimate and predict human motion. These predictions are integrated into an MPC controller which generates robot commands by enforcing safety through discrete-time control barrier function (DT-CBF), ensuring that the robot avoids collisions while remaining within navigable regions. The approach is validated through high-fidelity simulations and real-world experiments using the TIAGo mobile manipulator. The results demonstrate that integrating vision-based semantic data with geometric constraints significantly improves collision avoidance and success rates in cluttered, multi-room scenarios.

## 1 Introduction

Mobile robots are increasingly being deployed in human-populated environments for tasks such as delivery, surveillance, and assistance. These scenarios often require the robot to navigate through complex, dynamic spaces while ensuring safety by leveraging the knowledge of its own state and relying on sensor information. A key challenge is enabling the robot to reach predefined goals on a map, often located in different rooms, while avoiding both static obstacles and crowds. The ability to predict and respond to human motion in real-time settings is of utmost importance.

Recent advances in robot navigation emphasize the importance of integrating human motion prediction into control strategies to ensure safe (i.e., collision-free) and predictable trajectories, facilitating effective human–robot coexistence in shared environments (Martinez-Baselga et al., 2025; Gu et al., 2022; Salzmann et al., 2020; Muchen Sun et al., 2025; Paez-Granados et al., 2022; Samavi et al., 2024).

Accurately responding to human motion involves estimating their state (position and velocity) and predicting their trajectories over a finite time horizon. Classical approaches often represent humans as velocity obstacles (Fiorini and Shiller, 1998), where certain regions of the velocity space are deemed unsafe due to the risk of collision. The method has evolved into reciprocal interaction frameworks (Van den Berg et al., 2008; Van Den Berg et al., 2011) that account for mutual avoidance behaviors. Alternative models, such as social force formulations (Jafari and Liu, 2024), simulate human trajectories as the result of attractive and repulsive forces in the environment. These methods are computationally efficient and easily interpretable. However, recent findings suggest that the choice of human motion prediction model may not significantly alter the overall performance of the control system (Poddar et al., 2023).

To develop a successful crowd navigation framework, it is essential to understand the broader concept of autonomous navigation. This process can be structured into three interconnected layers: perception, cognition, and operation (de Carvalho et al., 2024). The perception layer is responsible for acquiring and interpreting environmental data. It allows the robot to sense its surroundings and localize itself within a map, often constructed in real time using simultaneous localization and mapping techniques (Henning et al., 2023; Panigrahi and Bisoy, 2022; Yang, 2012; Ling and Shen, 2017) leveraging onboard sensor data. Building upon this, the cognition layer integrates navigability information and performs high-level path planning, computing a feasible trajectory toward the goal. This layer often relies on deterministic or stochastic graph-based approaches, such as probabilistic roadmaps (Atas et al., 2021; Sgorbissa and Zaccaria, 2012), occupancy grid maps (Li et al., 2014; Esenkanova et al., 2013), or classical algorithms including Dijkstra (Niu et al., 2016; Casalino et al., 2009), to identify collision-free paths and determine whether certain regions are traversable. Finally, the operation layer converts the high-level plan into executable control inputs for the robot. This layer often employs model-based control strategies, such as model predictive control (MPC), which plans the motion of the robot over a finite time horizon. MPC enables goal-oriented or trajectory-oriented navigation while satisfying a broad set of constraints, including avoidance of static obstacles and humans while satisfying the kinematic limits of the robot (Atas et al., 2023; Mayne et al., 2000; Stefanini et al., 2024).

In this study, control inputs are generated by solving an MPC problem that leverages real-time sensor data and a known map of the environment to navigate through multi-room spaces, which are inherently non-convex. The perception and prediction of surrounding humans build upon the approach by Vulcano et al. (2022), employing finite state machines (FSMs) to dynamically regulate the operational state of the Kalman filters (KFs) used for tracking. To improve human detection, the system fuses data from both a LiDAR and an RGB-D camera, providing rich spatial and semantic information. For improving collision avoidance, the MPC formulation incorporates constraints based on the control barrier function (CBF) framework (Ames et al., 2019), ensuring that the robot maintains safe distances from both static obstacles and humans, building an additional layer of robustness to the overall system.

Beyond the technical aspects of navigation and obstacle avoidance, the framework addresses the challenges of scalability and computational efficiency. Human-populated environments are often characterized by rapidly changing conditions, including varying densities of moving humans and spatial configurations. By focusing on the closest humans, the estimation and control modules balance computational efficiency with real-time performance, allowing the system to operate seamlessly in crowded spaces and trying to limit the freezing robot problem (Trautman and Krause, 2010). Furthermore, the modularity of the approach enables easy adaptation to different robot platforms, sensor configurations, and planning algorithms.

**FIGURE 1:**
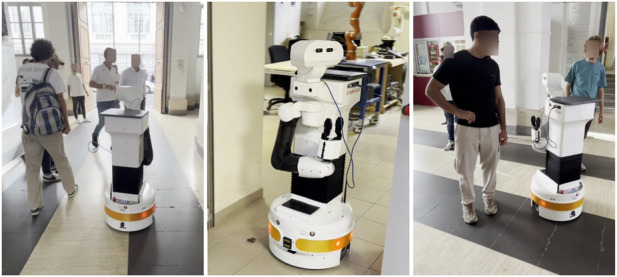
Snapshots of the TIAGo mobile manipulator, powered by the proposed sensor-based control framework, safely navigating multi-room environments populated by humans. Video: https://youtu.be/RYsQ3zmv30w.

This study details the design and implementation of the proposed complete framework, emphasizing the integration of high-level planning, perception, and motion generation. Simulations and experimental results highlight the impact of perception on overall performance and demonstrate the ability of the robot to navigate multi-room environments responding promptly to human motions (Figure 1).

The paper is structured as follows: Section 2 reviews related work in crowd navigation, human motion estimation and prediction, and semantic understanding using multiple sensors; Section 3 describes the formulation of the problem and related challenges; Section 4 introduces the approach proposed in this study; Section 5 details the map generation and the multi-room planning; Section 6 explains in detail the crowd detection, estimation, and prediction modules; Section 7 includes the MPC formulation with the obstacle avoidance; Section 8 outlines the experimental setup and results; Section 9 concludes the paper by introducing potential future directions.

## 2 Related work

Crowd-aware navigation is a critical challenge in autonomous robotics, particularly in indoor environments, where robots must reach goals across multiple rooms while avoiding collisions with walls, furniture, and humans. In the following, we review the most relevant approaches proposed to address this problem.

### 2.1 Crowd navigation strategies

Navigation in crowded spaces has been addressed from several complementary angles, ranging from reactive geometric rules and data-driven policies to optimization-based controllers with explicit safety certificates and, more recently, to sampling-based and search-based planners that reason about pedestrian uncertainty. Purely data-driven approaches, such as those using reinforcement learning or imitation learning, offer strong adaptability in unstructured environments but often lack interpretability, generalization, and safety guarantees, limiting their reliability in real-world deployments (Wen et al., 2024). Additionally, they require extensive training and large datasets to ensure robustness against sensor noise and environmental variability.

In contrast, optimization-based methods provide explicit formulations for safety and dynamic feasibility. For instance, Brito et al. (2019) used linear model predictive contouring control with KF-based obstacle tracking to ensure safe navigation around humans. Other studies proposed the joint optimization frameworks that simultaneously plan the robot trajectory and forecast human motion (Chen et al., 2021; Samavi et al., 2024; D’Orazio et al., 2025). Although these methods offer high accuracy and safety, they may suffer from high computational load, especially in dense scenarios.

To mitigate these limitations, hybrid strategies have emerged that integrate learning-based motion forecasting into model-based planning. For example, Le et al. (2024) used deep neural networks for predicting multi-modal human trajectories, which are then used as inputs to a planning module. Gupta et al. (2018) and Alahi et al. (2016) further demonstrated how generative models and recurrent neural networks can handle the inherent uncertainty and sequential nature of human movement. However, recent findings argue that more sophisticated prediction models do not always translate into better navigation outcomes when integrated into standard MPC frameworks (Poddar et al., 2023).

Another common issue in optimization-based systems is the risk of local minima due to limited planning horizons. To overcome this, hierarchical approaches are often adopted. A global planner determines a coarse path to the goal, which is then refined using a local controller that handles real-time collision avoidance (Alonso-Mora et al., 2017). Deep reinforcement learning has also been applied to infer intermediate goals or navigation way-points, guiding the optimization process (Wen et al., 2024; Brito et al., 2021). Alternatively, proactive avoidance strategies mitigate deadlocks, such as the approach proposed by Wei et al. (2025), which couples dynamic object tracking with a potential-field-based selection of avoidance points to steer the robot away from approaching humans before resuming its task.

Hybrid approaches rely on sampling-based and tree-search techniques, blending model-based safety guarantees with stochastic exploration of the action or belief space. For example, Gupta et al. (2022) formulated crowd navigation as an online partially observable Markov decision process solved via Monte Carlo tree search (MCTS), explicitly reasoning about pedestrian intention uncertainty, while Bonanni et al. (2025) combined MCTS with velocity obstacles to obtain safe plans in cluttered dynamic environments.

Methodologically closer to our work, Yang et al. (2019) and Majd et al. (2021) integrated CBFs into sampling-based planners to yield collision-free trajectories around static and moving obstacles, while Zhang et al. (2025) combined a convex decomposition of the environment with an optimization-based formulation enforcing CBF-based constraints; however, the former approaches are validated solely in simulation scenario, assuming known or predictable dynamics of obstacles, while the latter only considers static obstacles, leaving dynamic crowds outside the scope of that formulation.

Most of the cited works present critical limitations: (i) they assume open, obstacle-free spaces, neglecting the clutter and topological complexity of realistic multi-room environments, such as working and domestic areas; (ii) they assume that the full state of each human is available from an idealized sensing layer, relying either on external motion-capture systems (e.g., Vicon) or on ground-truth simulated data; and (iii) they use simplistic distance-based constraints for collision avoidance. We address these gaps by leveraging CBF within an MPC formulation, enabling robust safety constraints in crowded, multi-room environments with active perception based on the onboard sensors.

The abovementioned related studies can also be contrasted along three dimensions directly relevant to our problem. First, in terms of *environment representation*, they range from implicit free-space encodings used in reinforcement-learning and sampling-based planners (Brito et al., 2021; Yang et al., 2019; Majd et al., 2021; Gupta et al., 2022; Bonanni et al., 2025) to tracked-obstacle sets and potential fields used in MPC-based frameworks (Brito et al., 2019; Wei et al., 2025) and obstacle-wise convex decompositions (Zhang et al., 2025); in contrast, we explicitly build a topological graph of overlapping free-space convex regions that captures multi-room connectivity and provides safe transition gateways. Second, in terms of *planning hierarchy*, existing approaches span from single-layer reactive local controllers (Yang et al., 2019; Majd et al., 2021) to hierarchical schemes (Zhang et al., 2025; Brito et al., 2021) and tree-search-based planners (Gupta et al., 2022; Bonanni et al., 2025); our pipeline pairs a Dijkstra-based topological planner over convex regions with a receding-horizon local MPC. Third, in terms of *safety constraint formulation*, most studies rely on distance-based constraints (Brito et al., 2019; Le et al., 2024), velocity-obstacle pruning (Bonanni et al., 2025), and belief-space cost shaping (Gupta et al., 2022); similar to few other approaches (Vulcano et al., 2022; Majd et al., 2021), our formulation employs CBFs to avoid collision with both environment and crowd. It is the combination of these three ingredients—topological free-space decomposition, hierarchical global–local planning, and CBF-based safety constraints—that distinguishes the proposed framework from the prior art.

### 2.2 Sensor fusion for semantic perception

The perception system is the cornerstone of autonomous navigation. Many existing approaches use LiDAR for reliable geometric mapping and obstacle detection. For instance, Vulcano et al. (2022), Patle et al. (2018), and Yoon et al. (2018) used LiDAR data to inform the controller about obstacle proximity, enabling dynamic plan adaptation.

However, relying solely on laser data is insufficient in real-world experiments, where the environment is subject to changes not only from moving humans but also from “movable” static objects, such as chairs, trash bins, and tables. To enhance scene understanding, RGB-D cameras have also been integrated for semantic perception. Arreghini et al. (2025), Skoczeń et al. (2021), De Cristóforis et al. (2015), and Xiao et al. (2015) utilized object detection algorithms to identify and track humans. In our previous study (Carboni et al., 2024), we employed a YOLO-based detector (Redmon et al., 2016) on RGB-D images, enhancing the robustness of human tracking in cluttered and partially occluded environments.

In the proposed framework, we combine LiDAR and RGB-D information into a unified perception pipeline enabling both spatial accuracy and semantic richness, which are critical for human detection. The resulting crowd estimation feeds into an MPC for safe and efficient navigation in realistic indoor settings.

## 3 Problem formulation

**FIGURE 2:**
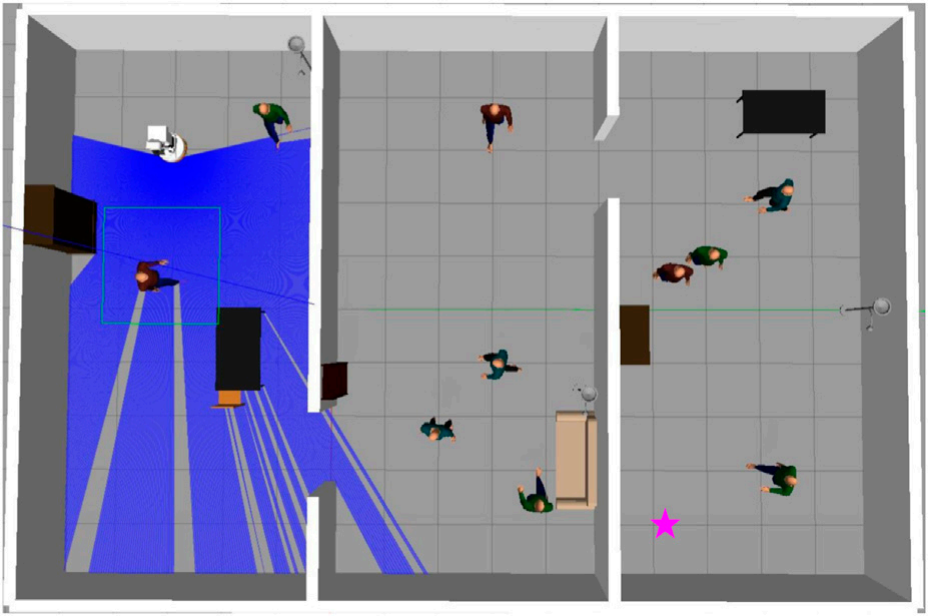
A representation of the addressed problem in a simulated three-room environment with 10 humans. The magenta star indicates the goal, while the blue rays are spanned by the LiDAR at the base of the robot.

Throughout this study, we consider a mobile robot, which must navigate a 3D environment W populated by static obstacles (e.g., walls and furniture), as well as humans (Figure 2).

To account for the kinematic constraints typical of mobile robots (e.g., nonholonomic constraints in wheeled robots), one defines the system state as x=(q,μ) , which includes both the n -dimensional configuration q and the m -dimensional pseudovelocity μ ; for example, refer Siciliano et al. (2025). The state evolution is governed by a nonlinear continuous-time dynamic model, which we assume to have been discretized with sampling time δ . At a generic time $t_{k}$ , the discrete-time model is then expressed as:

$$x_{k+1}=F\left(x_{k},u_{k}\right),$$ (1)

where $u_{k}$ is the control input applied to the system at $t_{k}$ .

Our aim is to design a real-time controller that drives the robot to an assigned goal while avoiding collisions. More formally, we can state the following objectives.

1. *Feasibility:* the robot moves consistently with its dynamic model, while respecting all the additional kinematic and actuation constraints, which are not already embedded in Equation 1.
2. *Safety:* the motion of the robot in W is collision-free in that both static obstacles and humans are avoided at all times.
3. *Navigation:* the robot position p , represented by the (x,y) coordinates of a specific point on its body (e.g., its geometric center), asymptotically converges to the target $p^{\text{goal}}$ .

Concerning the safety objective, it should be acknowledged that it is obviously impossible to guarantee collision avoidance in the presence of autonomous agents (the humans) which move of their own will, unpredictably, and may be reckless or even malicious. In principle, one could try to attack the problem by artificially imposing behaviors (e.g., collision aversion) or constraints (e.g., maximum velocity) on the human motion. However, we prefer to focus on the following, more realistic definition of safety: at each time instant, the future motion of the robot over a fixed control horizon *is predicted to be collision-free based on the currently available data*.

The new formulation of the safety objective emphasizes the role of the information used by the robot to take motion decisions. Such information refers to the humans and to the robot itself. As for the humans, the robot must estimate their state and predict their future trajectory based on the observations O gathered by its exteroceptive sensors, which we will assume to be a 2D laser rangefinder and an RGB-D camera. As for the robot, we have worked under the premise that a localization module provides a reliable estimate of its full state.

## 4 Proposed approach

**FIGURE 3:**
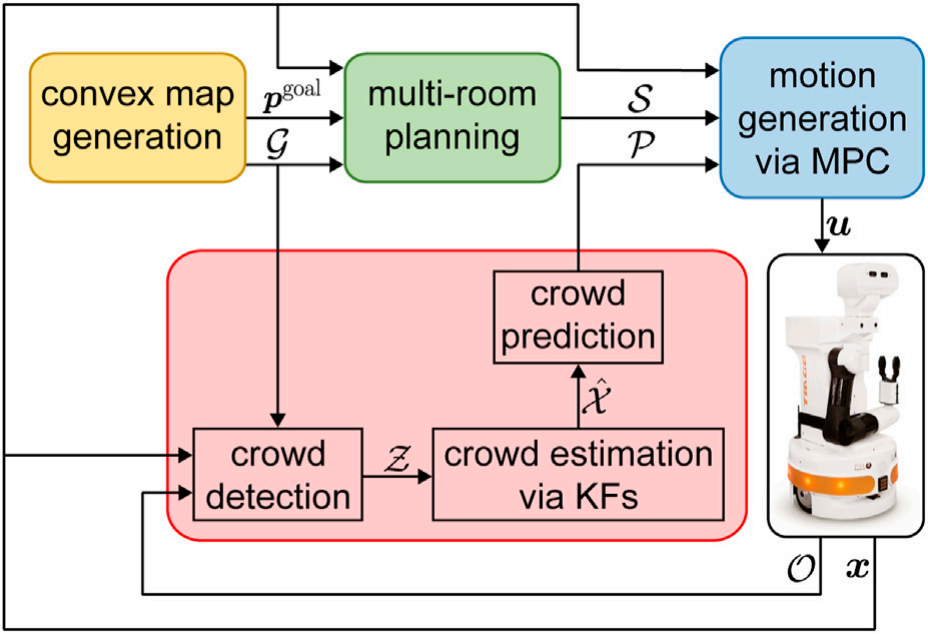
Block diagram of the proposed framework. The red block processes raw observations O to produce crowd motion prediction P via detection, estimation, and prediction modules. The yellow block decomposes the environment into a set of convex polytopes and constructs the graph G . The green module plans the sequence of regions S to reach the goal position $p^{\text{goal}}$ , and the blue module generates the control inputs u via an MPC. The robot is localized in the environment, and its state x is always known.

To address the problem formulated in Section 3, we propose a complete pipeline, which consists of four main blocks, as illustrated in Figure 3.

The convex map generation block processes the static map to decompose the free space into a set of overlapping convex polytopes called convex regions. This decomposition, which renders the problem tractable and the solving procedure efficient, transforms the continuous geometric map into a discrete topological graph G , where nodes represent convex regions and edges represent via points used for transitions. The multi-room planning block utilizes this graph G and the current state of the robot x to compute the optimal sequence of convex regions S that guides the robot toward the goal $p^{\text{goal}}$ . The framework allows the planner to operate online, enabling the dynamic recomputation of the region sequence in response to real-time changes, such as the assignment of a new goal position. Simultaneously, the perception block (red) handles the detection, state estimation, and motion prediction of surrounding humans. The crowd detection block processes raw observations $O=\left\{O^{\text{las}},O^{\text{cam}}\right\}$ , gathered via an onboard laser rangefinder and a RGB-D camera, to detect human positions. It collects them into a measurement set Z and sends them to the crowd estimation module, which employs KFs to estimate human states $\hat{X}$ . The crowd prediction module unrolls the future trajectories P of each individual estimated human.

Finally, the motion generation block synthesizes control inputs u . It executes a real-time MPC algorithm combining the high-level plan S with the crowd predictions P while enforcing CBFs constraints to ensure safe and efficient execution within the identified convex region.

## 5 Map generation and planning

### 5.1 Convex map generation

The convex map generation block abstracts the complex geometry of the environment into a discrete topological graph G=(R,V) , which is subsequently used by the planner to determine the sequence of rooms to traverse. The construction of this graph proceeds in three distinct steps: convex decomposition, via point selection, and graph construction.

#### 5.1.1 Convex decomposition

Given a planar map of the environment, we decompose its free space into a set of overlapping convex polytopes (i.e., convex regions), denoted as $R=\left\{R^{1},\ldots R^{n_{r}}\right\}$ . These regions can be defined manually, as done in our experimental setup, or computed via standard algorithms from the literature (Zhong et al., 2020; Deits and Tedrake, 2015a; Deits and Tedrake, 2015b). The proposed control framework is entirely independent of the map generation pipeline. The only requirements for R are the following:

- Each region $R^{j}$ , $j=1,\ldots ,n_{r}$ , must lie entirely within the obstacle-free subsets of the environment (this is needed in order to be able to guarantee safety with respect to static obstacles).
- Adjacent regions must have a feasible overlap (this is needed to ensure the connectivity of R ).

**FIGURE 4:**
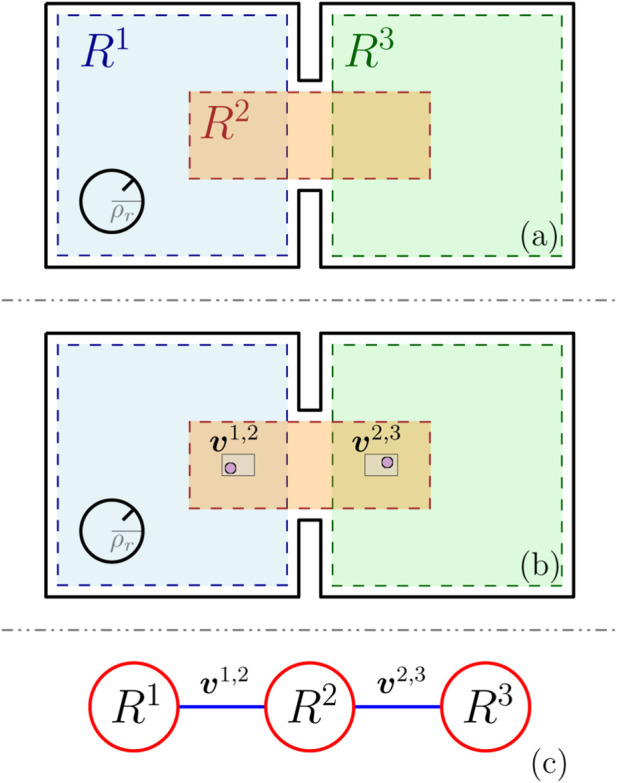
The three-stage process for constructing the topological graph. **(a)** Convex decomposition: the environment is decomposed into overlapping convex polytopes (e.g., rooms $R^{1}$ , $R^{3}$ , and transition region $R^{2}$ ). **(b)** Via point selection: for each overlap (e.g., $R^{1}\cap R^{2}$ ), the system checks for a via point $v^{1,2}$ (magenta dot) in the eligible subspace of points (black region) such that the robot footprint (circle of radius $\rho_{r}$ ) fits entirely within the intersection when it is on $v^{1,2}$ . **(c)** Graph construction: valid via points form the edges of the final graph G , where nodes correspond to regions. If the condition in **(b)** is not met, no edge is created, preventing the planner from selecting impassable routes.

For example, Figure 4a shows a possible environment with two rooms connected via a door. In the final decomposition, the region $R^{2}$ creates the necessary overlap between the two regions $R^{1}$ and $R^{3}$ which represent the rooms.

#### 5.1.2 Connectivity analysis and via point selection

Connectivity between any pair of adjacent regions $R^{j}$ and $R^{\ell }$ is not guaranteed by a simple overlap. An overlap is deemed feasible only if there exists a valid via point $v^{j,\ell }\in {I\;}^{j,\ell }$ , with ${I\;}^{j,\ell }=R^{j}\cap R^{\ell }$ , such that the robot, when positioned at $v^{j,\ell }$ , is fully contained within the intersection. Mathematically, we seek a point $v^{j,\ell }$ such that:

$$\text{dist}\left(v^{j,\ell },\partial {I\;}^{j,\ell }\right)\geq \rho_{r},$$ (2)

where $\rho_{r}$ denotes the radius of the robot footprint, modeled as a circular region, i.e., a bounding circle, including the robot projection onto the ground and an additional safety margin, and $\partial {I\;}^{j,\ell }$ represents the boundaries of the intersection polygon. Any point satisfying Equation 2 can be a candidate via point $v^{j,\ell }$ , which acts as a safe “gateway” to ensure that the robot satisfies the safety constraints of both regions simultaneously during the transition (Figure 4b). This geometric check serves as the formal verification procedure for the generated regions, ensuring that narrow passages and doorways are treated safely.

#### 5.1.3 Graph construction

Finally, the graph G is constructed (Figure 4c). The convex regions are chosen as the nodes of the graph. An edge is created between two nodes $R^{j}$ and $R^{\ell }$ if and only if a valid via point $v^{j,\ell }$ has been successfully identified in the previous step. If the intersection $I^{j,\ell }$ is too narrow to contain the robot footprint (i.e., no point satisfies Equation 2), no edge is created. This results in a disconnected graph component, correctly reflecting that the passage is physically impassable for the robot. The set of validated via points V is the edge set of the graph.

### 5.2 Multi-room planning

The goal of the multi-room planning block is to compute the optimal topological path S to guide the robot from its current location p to the goal $p^{\text{goal}}$ (detailed in Supplementary Algorithm 1).

Since the robot is localized within the map, the first step is to associate its position p with a discrete node in the graph G . We identify the candidate regions that fully contain the robot footprint; assuming at least one exists, we define the current region $R^{\text{curr}}$ as the candidate that minimizes the graph distance to the goal, thereby resolving any ambiguity when the robot is located within an intersection.

Similarly, we identify the goal region $R^{\text{goal}}$ as the convex polytope containing the target coordinates $p^{\text{goal}}$ . If $p^{\text{goal}}$ lies outside the aggregated navigable area $A={⋃}_{\ell =1,\ldots ,n_{r}}R^{\ell }$ , the goal region $R^{\text{goal}}$ is selected as the closest to $p^{\text{goal}}$ .

Once $R^{\text{curr}}$ and $R^{\text{goal}}$ are established, we employ the Dijkstra algorithm (Dijkstra, 2022) to compute the sequence of regions $S=\left\{R^{\text{curr}},\ldots ,R^{\text{goal}}\right\}$ that minimizes the cumulative distance traversed. S consists solely of region nodes; the associated via points required for the transition are automatically retrieved from the graph definition G in the motion generation block.

## 6 Perception pipeline

**FIGURE 5:**
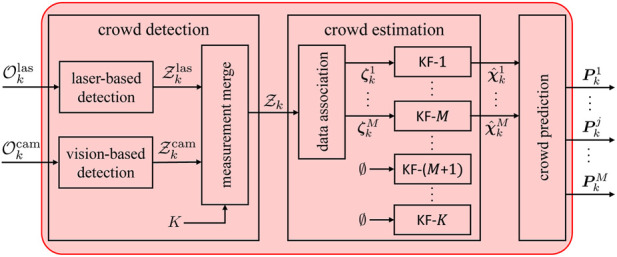
Conceptual scheme of the perception pipeline. The crowd detection block takes the raw observations from laser and camera and merges them into a unique measurement set $Z_{k}$ of at most K elements. Then, $Z_{k}$ is provided to the crowd estimation block, which associates the current measurements with past estimates and outputs the current estimates using a set of KFs.

With reference to Figure 5, at the generic time instant $t_{k}=k\delta$ , where δ denotes the sampling time, the perception block processes the available sensory information $O_{k}=\left\{O_{k}^{\text{las}},O_{k}^{\text{cam}}\right\}$ , obtained from the LiDAR and the camera, respectively. The output of this process is the set

$$P_{k}=\left\{P_{k}^{1},\ldots ,P_{k}^{M}\right\},$$

which contains the predicted trajectories of the M surrounding humans over the prediction horizon $\left[t_{k},t_{k}+T\right]$ , where T=Nδ and N is the number of prediction steps.

The predicted trajectory of the l -th human $P_{k}^{l}\in P_{k}$ is defined as:

$$P_{k}^{l}=\left({{\hat{p}}^{l}}_{0|k},\ldots ,{{\hat{p}}^{l}}_{N|k}\right),$$ (3)

where ${\hat{p}}_{i|k}^{l}$ denotes their predicted absolute position at future time $t_{k+i}=\left(k+i\right)\delta$ , derived from their state estimate ${\hat{p}}_{0|k}^{l}$ .

Since the number of humans detected using both sensors may be excessively large, potentially compromising real-time performance, we consider a maximum of K humans for collision avoidance as in Vulcano et al. (2022), ensuring that M≤K . The parameter K is chosen *a priori* based on an estimate of the crowd size and available computational resources.

The modules involved in the perception process are detailed in the following subsections.

### 6.1 Crowd detection

This module extrapolates relevant data from heterogeneous sensory information. The accuracy in distance estimation and the broad field of view (FOV) of a rangefinder are combined with the capability to extract visual context and semantic data from images obtained via an RGB-D camera. Due to the different sensor frequencies, $O_{k}^{\text{las}}$ and $O_{k}^{\text{cam}}$ are separately handled to rely on the latest available information.

#### 6.1.1 Laser-based detection

Since the laser observation $O_{k}^{\text{las}}$ consists of range-bearing pairs, it is convenient to first reconstruct the two-dimensional points in Cartesian coordinates. At this stage, points not contained in the navigable area A are filtered out. Later, the DBSCAN algorithm (Ester et al., 1996) is adopted for clustering, allowing the distinction between different obstacles. This density-based approach does not require a predefined number of clusters; it identifies clusters of arbitrary shapes, and it is robust to outliers. For each of the identified $n_{\text{clus}}$ clusters, we select its representative point considering two different strategies: taking the *closest* point to the robot within the cluster or choosing a virtual point obtained as the *average* of all the points in the cluster. Although the former approach is more conservative for safety purposes, we adopt the latter as it better accounts for cluster shape and avoids instantaneous change in the considered position due to the sensor noise. The resulting $n_{\text{clus}}$ representative points are collected into the laser measurement set

$$Z_{k}^{\text{las}}=\left\{z_{k}^{\text{las},1},\ldots ,z_{k}^{\text{las},n_{\text{clus}}}\right\}.$$

The laser-based detection procedure is reported in Supplementary Algorithm 2.

#### 6.1.2 Vision-based detection

The camera observation $O_{k}^{\text{cam}}$ provides RGB and depth images. Unlike laser-based detection, which detects every object within its FOV, the vision-based detection module uses the YOLO-v8 model (Jocher et al., 2023) to detect only the humans in the camera image. YOLO-v8 is a deep neural network trained on the COCO dataset (Lin et al., 2014), which processes the RGB image and outputs bounding boxes around detected humans. For each bounding box, we extract the center $c_{k}^{l}$ and a depth value $d_{k}^{l}$ , computed from the depth image. The Cartesian position of each human $z_{k}^{l}\in R^{2}$ is reconstructed using the pinhole camera model (Hartley and Zisserman, 2003) as follows:

$$z_{k}^{l}=\Pi_{xy}\left(R_{k}^{c}d_{k}^{l}K^{-1}c_{k}^{l}+p_{k}^{c}\right),$$

where $R_{k}^{c}$ and $p_{k}^{c}$ are the orientation and position of the camera, respectively, in the world frame at instant k , $\Pi_{xy}$ is a function extracting the x and y components of the argument, and K is the camera matrix, defined as follows:

$$K=\left(\begin{array}{ccc} f_{x} & 0 & c_{x}\\ 0 & f_{y} & c_{y}\\ 0 & 0 & 1 \end{array}\right),$$

where $\left(f_{x},f_{y}\right)$ is the focal lengths and $\left(c_{x},c_{y}\right)$ is the principal point offset. Points outside A are discarded, and the remaining $n_{c}$ positions are collected into the camera measurement set

$$Z_{k}^{\text{cam}}=\left\{z_{k}^{\text{cam},1},\ldots ,z_{k}^{\text{cam},n_{c}}\right\}.$$

The vision-based detection procedure is described in Supplementary Algorithm 3.

#### 6.1.3 Measurement merging

**FIGURE 6:**
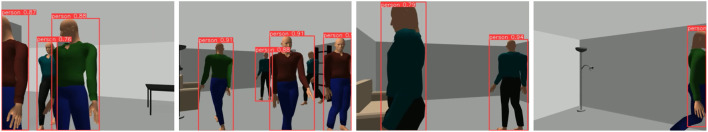
Snapshots of human detection from the onboard RGB-D camera using the YOLOv8 model in a simulation environment. The model demonstrates robust performance in identifying humans even under challenging conditions, including partial occlusions and crowded scenes.

Since the FOVs of the two sensors may overlap, detections of the same human could be present in both $Z_{k}^{\text{las}}$ and $Z_{k}^{\text{cam}}$ . We exploit this redundancy to improve localization accuracy by fusing associated detections through position averaging. Detection association is performed using a distance-based strategy. We assume that the planar projection of the l -th human can be enclosed within a circular region of radius $\rho_{h}$ , referred to as the human footprint. A detection from $Z_{k}^{\text{las}}$ and one from $Z_{k}^{\text{cam}}$ are considered to correspond to the same human if they are mutual nearest neighbors and their distance is less than $\rho_{h}$ . Leveraging visual data is essential to mitigate ambiguities inherent to laser-based human detection. When multiple humans are in close proximity, laser observations may be merged into a single cluster, preventing correct individual identification. Conversely, vision-based detectors exploit appearance cues and are able to distinguish several humans even at very close distances, as clearly illustrated in Figure 6, thereby providing separate measurements for each individual. To address situations where the vision system identifies multiple individuals corresponding to a single laser cluster, we pair the laser measurement with the closest camera detection, while the remaining visual detections are treated as distinct, unpaired humans. We populate the final combined set $Z_{k}$ of size M≤K , by merging paired detections into their mean position and including unpaired detections, prioritizing those closest to the robot until all measurements have been processed or the size limit K is reached.

### 6.2 Crowd estimation via Kalman filters

This module deals with state estimation of the most dangerous M humans associating each one with a KF, referred to as KF-1, …, KF- M . The humans are free to move in all directions, but their velocity is assumed to be constant. The state of the l -th human at time $t_{k}$ is defined as $\chi_{k}^{l}=\left(p_{k}^{l},{\dot{p}}_{k}^{l}\right)$ and is estimated by KF- l using the following system dynamics and measurement models:

$${\hat{\chi }}_{k+1}^{l}=\left(\begin{array}{cc} I_{2} & \delta I_{2}\\ 0_{2\times 2} & I_{2} \end{array}\right){\hat{\chi }}_{k}^{l}+v_{k},$$ (4)

$${\zeta^{l}}_{k}=\left(\begin{array}{cc} I_{2} & 0_{2\times 2} \end{array}\right){\hat{\chi }}_{k}^{l}+w_{k},$$ (5)

where $I_{n}$ stands for the n×n identity matrix; $v_{k},w_{k}$ are white Gaussian noises with zero mean and covariance matrices $V_{k},W_{k}$ , respectively; ${\zeta^{l}}_{k}$ is the measurement associated with the l -th human and extracted from $Z_{k}$ .

#### 6.2.1 Data association

To maintain continuity in state estimation, data association is performed between current measurements and past estimates. Specifically, this ensures that as long as a human remains among the accounted M , it continues to be tracked by the same KF. The association is carried out using a closest-distance approach based on the Mahalanobis distance:

$$d^{m,l}={\left({\left(z_{k}^{m}-{\hat{p}}_{k}^{l}\right)}^{T}\Omega_{k}^{l}\left(z_{k}^{m}-{\hat{p}}_{k}^{l}\right)\right)}^{\frac{1}{2}}$$ (6)

between the m -th measurement $z_{k}^{m}\in Z_{k}$ and the position estimate ${\hat{p}}_{k}^{l}$ provided by the KF- l , with information matrix of the position estimate denoted as $\Omega_{k}^{l}$ . Similar heuristics as in Section 6.1.3 are applied to prevent incorrect associations. We denote by ${\zeta^{l}}_{k}$ the j -th measurement $z_{k}^{j}\in Z_{k}$ that has been associated with the l -th KF by Equation 6, while an empty measurement ζ=∅ is assigned to the remaining KFs.

#### 6.2.2 State estimation

At each time step $t_{k}$ , the KF- l operates based on its previous state estimate ${\hat{\chi }}_{k-1}^{l}$ and the measurement ${\zeta^{l}}_{k}$ it receives, which can either be valid ( ${\hat{\chi }}_{k-1}^{l}\neq \emptyset$ and ${\zeta^{l}}_{k}\neq \emptyset$ ) or be empty ( ${\hat{\chi }}_{k-1}^{l}=\emptyset$ and ${\zeta^{l}}_{k}=\emptyset$ ). As in Vulcano et al. (2022), the KF behavior is regulated by an FSM based on the availability of measurements and the progression of time. The FSM has four distinct states to ensure proper operation and management of the state estimates:

– *Idle.* In this state, the KF is inactive, and no estimation process is running. The FSM transitions to the *Start* state as soon as a new measurement is received.
– *Start.* This state initializes the KF. If a new measurement is available, the KF initializes the state estimate and transitions to the *Active* state. Conversely, the FSM reverts to the *Idle* state.
– *Active.* In this state, the KF continuously updates the state estimates using incoming measurements as long as they are available. If measurements stop arriving, the FSM transitions to the *Hold* state.
– *Hold.* In this state, the KF temporarily updates the estimates using the last available measurement. The FSM transitions back to the *Active* state if a new measurement becomes available. However, if new measurements do not arrive within a predefined time limit, the FSM transitions to the *Idle* state, and the current estimate is discarded.

This FSM structure ensures that the KF remains computationally efficient by discarding stale estimates, responsive to measurement availability for rapid reactions, and robust to occlusions by handling temporary sensor data loss.

### 6.3 Crowd prediction

Given the set ${\hat{X}}_{k}=\left\{{\hat{\chi }}_{k}^{1},\ldots ,{\hat{\chi }}_{k}^{M}\right\}$ of state estimates provided by the estimation layer, where the state estimate of the l -th human is denoted as ${\hat{\chi }}_{k}^{l}=\left({\hat{p}}_{k}^{l},{\hat{\dot{p}}}_{k}^{l}\right)\neq \emptyset$ .

To predict the trajectory $P_{k}^{l}$ over the control horizon $\left[t_{k},t_{k}+T\right]$ , we assume a constant-velocity model for each human. Accordingly, the predicted position ${\hat{p}}_{i|k}^{l}$ at the future step i (corresponding to time $t_{k+i}$ ) is computed as follows:

$${\hat{p}}_{i|k}^{l}={\hat{p}}_{k}^{l}+i\delta {\hat{\dot{p}}}_{k}^{l},\forall i=0,\ldots ,N,$$

where δ is the sampling time and N is the number of prediction steps. The resulting collection of predicted trajectories $P_{k}^{l}=\left({\hat{p}}_{0|k}^{l},\ldots ,{\hat{p}}_{N|k}^{l}\right)$ is then forwarded to the motion generation block to enforce collision avoidance constraints.

## 7 Motion generation

The primary objective of this module is to synthesize the control input u that drives the robot toward the goal $p^{\text{goal}}$ while ensuring safety. Navigating a multi-room environment presents a significant challenge for real-time control as the global workspace is inherently non-convex.

To address this, we leverage the hierarchical structure of our framework. Although the multi-room planner handles the global complexity by determining the topological sequence of regions S , the motion generator solves a local optimization problem constrained within the current region $R^{\text{curr}}$ . Representing the free space as a set of convex polytopes is advantageous for the optimization solver. It allows us to formulate safety boundaries as sets of linear inequality constraints, which are computationally efficient to evaluate and significantly faster to solve compared to non-convex distance-based constraints (Lorenzen et al., 2025; Lee, 2025).

To navigate through regions, the MPC tracks a discrete sequence of via points rather than a continuous global path or reference trajectory. We chose this strategy over traditional continuous path tracking because rigid path adherence can be overly constraining in dynamic, human-populated environments. Via points act as necessary transit gateways, granting the MPC the freedom to safely deviate and avoid unpredictable elements without being penalized for leaving a predefined path. Our primary objective is successfully reaching the goal while leaving sufficient spatial autonomy to the robot.

The control problem is formulated as a regulation task, where the robot is commanded to reach a temporary target $p^{\text{des}}$ . Initially, $p^{\text{des}}$ is set to the via point $v^{\text{curr},\ell }$ connecting the current region $R^{\text{curr}}$ to the next region $R^{\ell }$ in the sequence S . The condition for switching this target is geometric rather than convergence-based. The reference $p^{\text{des}}$ is updated to the next via point, and $R^{\text{curr}}$ is set to $R^{\ell }$ , as soon as the robot footprint is entirely contained within $R^{\ell }$ to promote a smooth and continuous motion.

This process repeats iteratively until the sequence S is empty. At that point, $p^{\text{des}}$ is finally set to the global goal $p^{\text{goal}}$ . The robot motion concludes when the positioning error $\left\|e\|=\|p^{\text{des}}-p\right\|$ falls below a specified tolerance $\bar{e}$ . Whenever no feasible solution can be found by the MPC algorithm, the safest command is an emergency stop.

At each control cycle, the MPC solves a finite-dimensional nonlinear programming (NLP) problem using the kinematic model in Equation 1 along the prediction horizon T of N steps, the same considered in Section 6.3.

Denote the decision variables at $t_{k}$ by

$$\begin{array}{ll} & \Xi_{k}=\left(x_{0|k},\ldots ,x_{N|k}\right)\\ & U_{k}=\left(u_{0|k},\ldots ,u_{N-1|k}\right), \end{array}$$

where $x_{i|k}$ and $u_{i|k}$ are the predicted robot state and control inputs at $t_{k+i}$ . Let $p_{i|k}$ be the predicted robot position at $t_{k+i}$ , $p^{\text{des}}$ be the desired position, and $e_{i|k}=p^{\text{des}}-p_{i|k}$ be the predicted task error at $t_{k+i}$ . Then, the running and terminal cost are:

$$\begin{array}{cc} J_{i|k}\left(x_{i|k},u_{i|k}\right)= & \;e_{i|k}^{T}Qe_{i|k}+\mu_{i|k}^{T}R\mu_{i|k}+u_{i|k}^{T}Su_{i|k}\\ J_{N|k}\left(x_{N|k}\right)= & \;e_{N|k}^{T}Q_{N}e_{N|k}+\mu_{N|k}^{T}R_{N}\mu_{N|k}, \end{array}$$ (7)

where Q , R , and S are the weighting matrices of appropriate dimensions for the predicted task error, the robot driving, and steering velocities and the control effort along the prediction horizon, respectively; $Q_{N}$ and $R_{N}$ are weighting matrices for the predicted task error^1^ and the robot velocities at the final time instant, respectively.

The NLP problem can be formulated as:

$$\begin{array}{ll} \underset{\Xi_{k},U_{k}}{\min}\overset{N-1}{\underset{i=0}{\sum }} & J_{i|k}\left(x_{i|k},u_{i|k}\right)+J_{N|k}\left(x_{N|k}\right), \end{array}$$ (8a)

$$\begin{array}{l} \text{subject to:} \end{array}$$

$$\begin{array}{ll} & x_{0|k}-x_{k}=0, \end{array}$$ (8b)

$$\begin{array}{ll} & x_{i+1|k}-F\left(x_{i|k},u_{i|k}\right)=0\forall i\in I_{0}^{N-1}, \end{array}$$ (8c)

$$\begin{array}{ll} & x_{\min}\leq x_{i|k}\leq x_{\max}\forall i\in I_{0}^{N}, \end{array}$$ (8d)

$$\begin{array}{ll} & u_{\min}\leq u_{i|k}\leq u_{\max}\forall i\in I_{0}^{N-1}, \end{array}$$ (8e)

$$\begin{array}{ll} & \text{convex region constraints}, \end{array}$$ (8f)

$$\begin{array}{ll} & \text{collision avoidance constraints}, \end{array}$$ (8g)

where $I_{0}^{N}=\left\{0,1,\ldots ,N\right\}$ . Equation 8b sets the initial state; Equation 8c constrains the state to evolve following the system dynamics; Equations 8d, e enforce the state and input bounds, respectively; Equation 8f limits the motion of the robot within the bounds of the current convex region, and Equation 8g allows the robot to safely navigate avoiding humans. Once the NLP at $t_{k}$ is solved, only the first control sample $u_{0|k}$ is extracted from $U_{k}$ and applied to the robot. Supplementary Algorithm 4 details the overall procedure.

### 7.1 Convex region constraints

At each time step $t_{k}$ , we enforce that the robot footprint lies within $R^{\text{curr}}$ throughout the prediction horizon, where $R^{\text{curr}}$ is a convex polytope characterized by μ vertices ordered counterclockwise $\left\{m^{1},\ldots ,m^{\mu }\right\}$ . The boundaries are characterized by the set of inward-pointing normal vectors $\left\{n^{1},\ldots ,n^{\mu }\right\}$ , where $n^{j}$ corresponds to the edge connecting $m^{j}$ and $m^{j+1}$ (i.e., $n^{\mu }$ is associated with $m^{1}$ and $m^{\mu }$ ), with $j\in I_{1}^{\mu }$ .

The robot state x is considered safe with respect to the boundaries of the convex region if its footprint is contained within the region, i.e.,

$$\left(p-m^{j}\right)n^{j}\geq \rho_{r},\;\forall j\in I_{1}^{\mu }.$$

Since p∈x , we can define a set of safe states, the *safe set*, in which the robot does not violate the boundaries as the upper level set of a function $h_{s}\left(x\right)$

$$C^{s}=\left\{x:h_{s}\left(x\right)\geq 0\right\},$$

where

$$h_{s}\left(x\right)=\left(\begin{array}{c} h_{s}^{1}\left(x\right)\\ \vdots \\ h_{s}^{\mu }\left(x\right) \end{array}\right)=\left(\begin{array}{c} \left(p-m^{1}\right)n^{1}-\rho_{r}\\ \vdots \\ \left(p-m^{\mu }\right)n^{\mu }-\rho_{r} \end{array}\right).$$ (9)

In principle, enforcing $h_{s}\left(x\right)\geq 0$ for any state x , is a necessary and sufficient condition to avoid collision between the robot footprint and the boundaries. However, a purely distance-based constraint affects the optimization only when the obstacle is reachable by the robot within the prediction horizon, eventually resulting in late reactions (Vulcano et al., 2022). A more robust approach consists of using discrete-time control barrier functions (DT-CBFs) to formulate constraints to control the rate of change in Equation 9. Specifically, the function $h_{s}\left(x\right)$ is a DT-CBF if it satisfies:

$$h_{s}\left(x_{k+1}\right)\geq \left(I_{\mu }-\Gamma_{s}\right)h_{s}\left(x_{k}\right),$$ (10)

where $\Gamma_{s}=\gamma_{s}I_{\mu }$ and $0<\gamma_{s}\leq 1$ . By enforcing Equation 10, we impose an upper bound on the rate of decay of each function, ensuring a more conservative behavior near the boundaries of the safe set. Over the prediction horizon, we can express the DT-CBF-based constraints in Equation 8f as follows:

$$h_{s}\left(x_{i+1|k}\right)\geq \left(I_{\mu }-\Gamma_{s}\right)h_{s}\left(x_{i|k}\right),\;\forall i\in I_{0}^{N-1}.$$

### 7.2 Collision avoidance constraints

DT-CBF-based constraints are also formulated to prevent collisions with humans. Unlike Equation 9, which depends solely on the state of the robot, in this case we must also account for the state of each human to properly ensure safety. To this end, the DT-CBF-based constraint is defined with respect to an augmented state ${\tilde{x}}^{l}=\left(x,{\hat{p}}^{l}\right)$ , defined by combining the robot position with the available estimate of the l -th position of the human ${\hat{p}}^{l}$ . We consider the combined state to be safe if the robot and the human footprints do not overlap, centered at p and ${\hat{p}}^{l}$ with radii $\rho_{r}$ and $\rho_{h}$ , respectively. Accordingly, the safe set defined with respect to the l -th human is

$$C^{l}=\left\{{\tilde{x}}^{l}:h_{d}^{l}\left({\tilde{x}}^{l}\right)\geq 0\right\},$$

which is the super-level set of the function

$$h_{d}^{l}\left({\tilde{x}}^{l}\right)=\|p-{\hat{p}}^{l}{\|}^{2}-{\left(\rho_{r}+\rho_{h}\right)}^{2}.$$

$h_{d}^{l}\left({\tilde{x}}^{l}\right)$ is a DT-CBF if it satisfies:

$$h_{d}^{l}\left({\tilde{x}}_{k+1}^{l}\right)\geq \left(1-\gamma_{d}\right)h_{d}^{l}\left({\tilde{x}}_{k}^{l}\right),$$ (11)

with $0<\gamma_{d}\leq 1$ . By enforcing Equation 11 for M humans considering their predicted positions along the MPC horizon, we can formulate the DT-CBF-based constraints in Equation 8g as follows:

$$h_{d}^{j}\left({{\tilde{x}}_{i+1|k}}^{j}\right)\geq \left(1-\gamma_{d}\right)h_{d}^{j}\left({\tilde{x}}_{i|k}^{j}\right),\;i\in I_{0}^{N-1},\;j\in I_{1}^{M},$$

with ${\hat{p}}_{i|k}^{l}$ and ${\hat{p}}_{i+1|k}^{l}$ extracted from the element $P_{k}^{j}$ of $P_{k}$ .

## 8 Results

The proposed framework was implemented in Python with acados (Verschueren et al., 2022) to generate the C-code for the MPC solver, using a real-time iteration scheme (Diehl et al., 2002; Gros et al., 2020). The evaluation strategy is two-fold: extensive high-fidelity simulations in Gazebo with randomized initial human configurations were conducted to assess generalization and robustness across diverse scenarios, while real-world experiments using the robot TIAGo from PAL Robotics validated practical applicability and hardware viability. All simulations and experiments were conducted on an Intel Core i7-13650HX CPU (2.6 GHz) and an NVIDIA GeForce RTX 4060 GPU. The video showcasing sample simulations and experiments is available at https://youtu.be/RYsQ3zmv30w.

### 8.1 Implementation details and parameters

#### 8.1.1 Robot model

**FIGURE 7:**
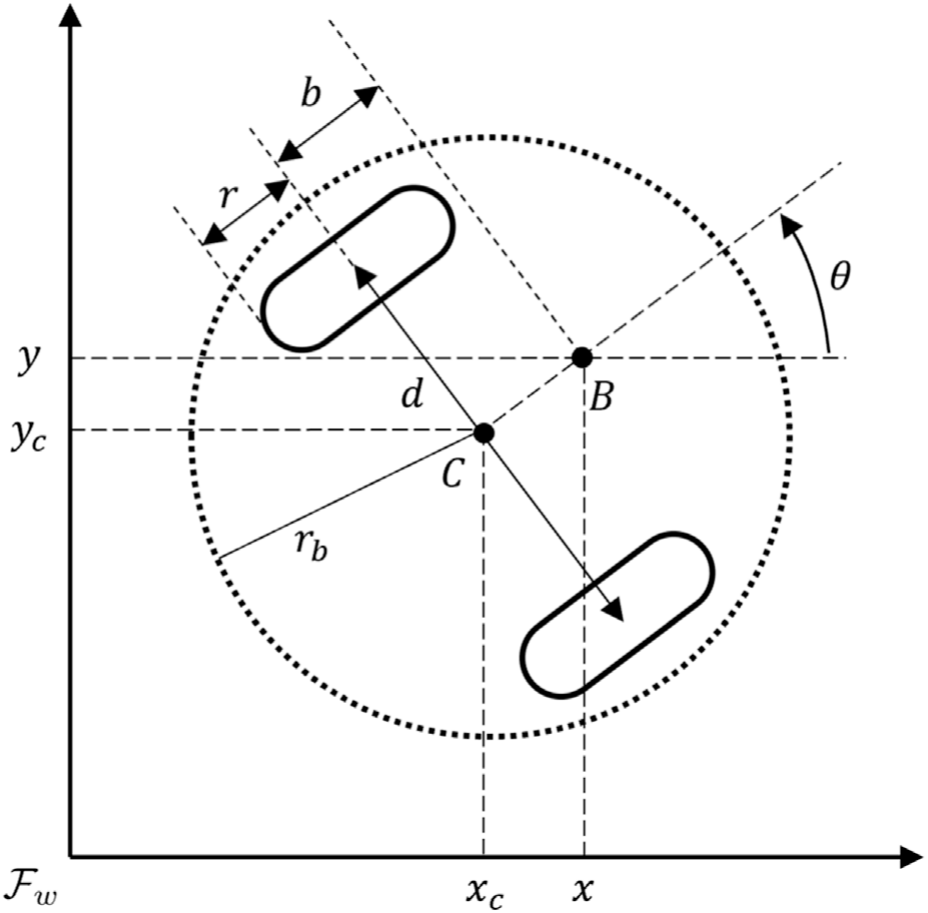
Model of the robot. $r_{b}$ is the radius of the mobile base of the robot, r is the radius of each wheel, d is the semi-axle length, and b is the distance between the center of the mobile base and the point (x,y) located along its sagittal axis.

The base of TIAGo is a differential-drive mobile robot, whose model is kinematically equivalent to a unicycle (Siciliano et al., 2025). With reference to Figure 7, the configuration vector is defined as the position and orientation of the robot q=(x,y,θ)∈SE[2] . The vector p=(x,y) represents the position of the point B located along the sagittal axis of the robot at a distance b>0 from its center C , while θ denotes the heading angle defined with respect to the x -axis of the world. The state of the robot is denoted as x=(q,μ) , where μ=(v,ω) collects the driving and steering velocities. By taking the angular accelerations of the right and left wheels as control inputs, $u=\left({\dot{\omega }}^{R},{\dot{\omega }}^{L}\right)$ , and assuming no wheel slippage, the kinematic model of the robot is defined as:

$$\dot{x}=f\left(x,u\right)=\left(\begin{array}{c} v\cos\theta -\omega b\sin\theta \\ v\sin\theta +\omega b\cos\theta \\ \omega \\ \frac{r}{2}\left({\dot{\omega }}^{R}+{\dot{\omega }}^{L}\right)\\ \frac{r}{d}\left({\dot{\omega }}^{R}-{\dot{\omega }}^{L}\right) \end{array}\right),$$ (12)

where r=0.0985 m is the wheel radius and d=0.4044 m is the track width. The forward offset is set to b=0.1 m.

#### 8.1.2 Algorithm parameters

The multi-room planning block operates at 1 Hz, enabling the system to adapt to real-time changes in the goal position or the convex map decomposition. Although the framework supports dynamic updates, in this study, the convex regions are manually defined and remain static. For the low-level control loop, the perception and motion generation modules are synchronized to the 15 Hz rate of the LiDAR (with the camera running at 30 Hz), resulting in a sampling time of δ=0.067 s.

The MPC prediction horizon is set to T=2.8 s, providing a sufficient look-ahead of N=42 steps. To balance safety and computational load, we track a maximum of K=5 humans. The CBF decay rates for Equations 10, 11 are set to $\gamma_{s}=\gamma_{d}=0.1$ .

For collision avoidance, the human footprint has a radius $\rho_{h}$ of 0.5 m in simulations and 0.35 m in experiments, while the robot footprint has a radius $\rho_{r}=r_{b}+b+0.01=0.38$ m centered in B (see Figure 7), where $r_{b}=0.27$ m is the radius of the robot base. When the MPC becomes infeasible due to deadlocks or sensor noise, we temporarily relax the safety constraints by reducing the radius to $\rho_{r}=r_{b}+0.01=0.28$ m and shifting the footprint center to the geometric center C to facilitate evasive maneuvers until the robot re-establishes a sufficient safety distance from the closest human.

#### 8.1.3 Sensor-aware cost function

The TIAGo robot is equipped with a SICK TIM571 LiDAR with a range of 25 m and a 180° FOV ( 220° in simulations) and an Xtion Pro Live RGB-D camera, featuring a 60° horizontal FOV and a maximum depth range of 8 m, both mounted facing forward. Mounted on a pan-tilt platform, the camera dynamically adjusts its orientation relative to the x -axis to point toward the nearest detected human to provide a more robust detection. Notably, when no surrounding obstacles are detected, the gaze of the camera is oriented toward the target position. Considering the horizontal rotation of the pan-tilt platform, the camera can span a FOV of 180° in total.

The sensors mounted facing forward create a blind spot behind the robot. To ensure safety, it is crucial that the robot prefers forward motion, keeping the goal and obstacles within its sensor field of view. To enforce this behavior, we modified the standard MPC cost function in Equation 7 by introducing a term that penalizes misalignment between the heading of the robot and the direction of the error vector. Let $n_{i|k}=\left(\cos\theta_{i|k},\sin\theta_{i|k}\right)$ be the sagittal projector along the robot forward direction at step $t_{k+i}$ . Then, the running and terminal cost become

$$\begin{array}{ll} & {\tilde{J}}_{i|k}\left(x_{i|k},u_{i|k}\right)=J_{i|k}\left(x_{i|k},u_{i|k}\right)-e_{i|k}^{T}Hn_{i|k}\\ & \;{\tilde{J}}_{N|k}\left(x_{N|k}\right)=J_{N|k}\left(x_{N|k}\right)-e_{N|k}^{T}H_{N}n_{N|k}, \end{array}$$

where matrix H is a weighting matrix of suitable dimension. The term $-e_{i|k}^{T}Hn_{i|k}$ rewards configurations where the robot faces the target ( $e_{i|k}$ and $n_{i|k}$ are aligned), effectively discouraging backward motion.

### 8.2 Simulations

To validate the proposed method and assess the impact of different perception modalities, we conducted extensive simulations in Gazebo.

#### 8.2.1 Validation of the human state estimation

**FIGURE 8:**
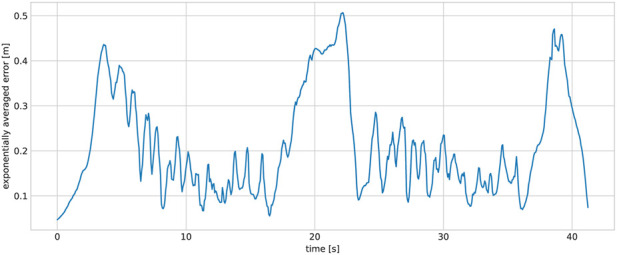
Prediction error $\epsilon_{k}$ for a simulated scenario in which a human moves back and forth along a line at 0.7 m/s. At each time k , $\epsilon_{k}$ is the weighted sum of the per-step errors between the predicted human positions ${\hat{p}}_{i|k}$ and the corresponding ground-truth positions $p_{k+i}$ , computed over the 42-step horizon and exponentially weighted to favor earlier instants in the control horizon. Error peaks coincide with abrupt motion changes (starting, stopping, and reversing direction), while low-error regions correspond to steady, constant-velocity phases.

Under the constant-velocity assumption, the quality of the predicted human positions depends entirely on the state estimation performance of the KF. To assess the responsiveness of the filter to unpredictable motion changes, we evaluated a simulated scenario in which a human moves back and forth along a segment at a constant velocity of 0.7 m/s, in front of a stationary robot and always within the sensor range. At each time instant k , we compute the per-step prediction error $\epsilon_{i|k}=||{\hat{p}}_{i|k}-p_{k+i}||$ between the predicted human position ${\hat{p}}_{i|k}$ at the i -th step of the horizon and the corresponding ground-truth future position $p_{k+i}$ . We then aggregate these errors into a single scalar $\epsilon_{k}=\frac{\sum_{i}w_{i}\epsilon_{i|k}}{\sum_{i}w_{i}}$ using exponentially decaying weights $w_{i}=\exp\left(\frac{-2i}{N-1}\right)$ , i=0,…,N−1 , with N=42 . This exponential biasing favors accuracy at short look-ahead times: since the MPC resolves the optimal control problem at every timestep, prediction inaccuracies at the far edges of the horizon have a minimal impact on the immediate safety of the robot. Figure 8 reports $\epsilon_{k}$ over time. As expected, the largest errors coincide with sudden motion changes, namely when the human initiates or halts movement or reverses direction, while the predictions remain highly accurate throughout the constant-velocity phases.

#### 8.2.2 Overall framework evaluation

**FIGURE 9:**
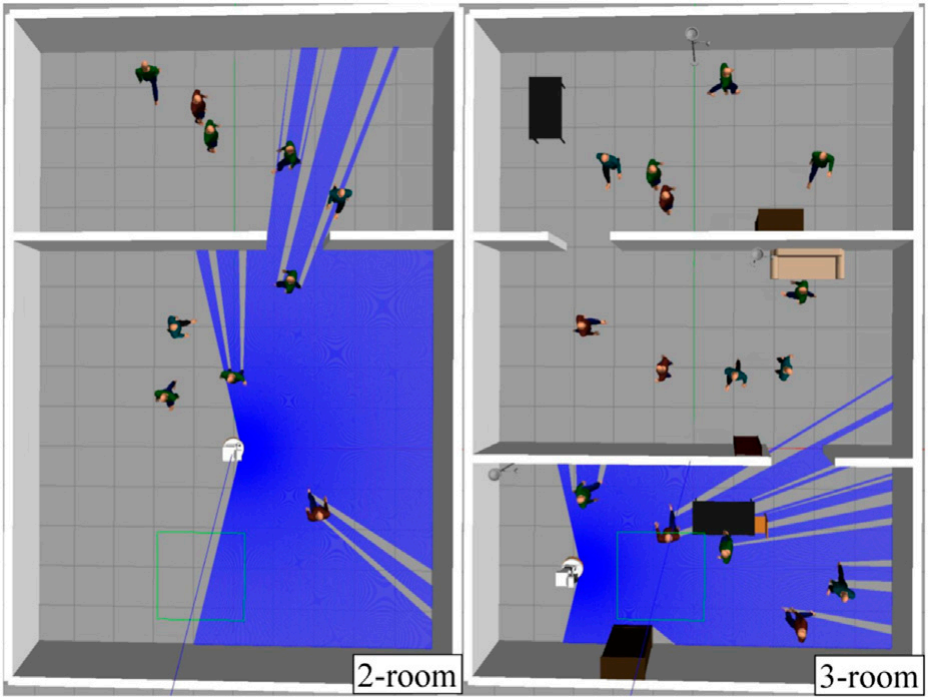
Simulation environments: (left) A two-room scenario with 10 humans; (right) A three-room scenario populated with 15 humans and furniture. In both settings, humans exhibit heterogeneous behaviors: some remain stationary, while others move following straight paths, creating dynamic and realistic scenarios.

To evaluate the performance of the framework, we varied the environment layout (two-room vs. three-room as in Figure 9) and the crowd density ( $n_{h}$ = 10 or 15). For each combination, we generated 20 randomized scenarios by sampling the robot initial configuration, the goal position, and the human trajectories. Each scenario was then executed twice, once relying solely on laser perception, and once combining laser and camera perception, resulting in a total of 160 runs. For each scenario, the robot initial configuration and goal position were randomly sampled, ensuring a minimum distance of 8 m between them. Human trajectories were randomly generated with a maximum walking speed of 0.5 m/s, following these rules: (i) humans could either be moving or be standing still; (ii) between 10 % and 40 % of the humans were spawned in pairs, either walking or standing, to reflect real-world situations where people often form small groups; (iii) standing humans were not spawned in close proximity to the robot, at the goal position, or near narrow passages (e.g., door frames), to avoid creating unsolvable navigation scenarios. Importantly, the crowd was non-cooperative, with humans being unaware of the robot presence.

We discuss in detail three representative simulations that illustrate the performance of the control framework and the key role of sensor fusion.

**FIGURE 10:**
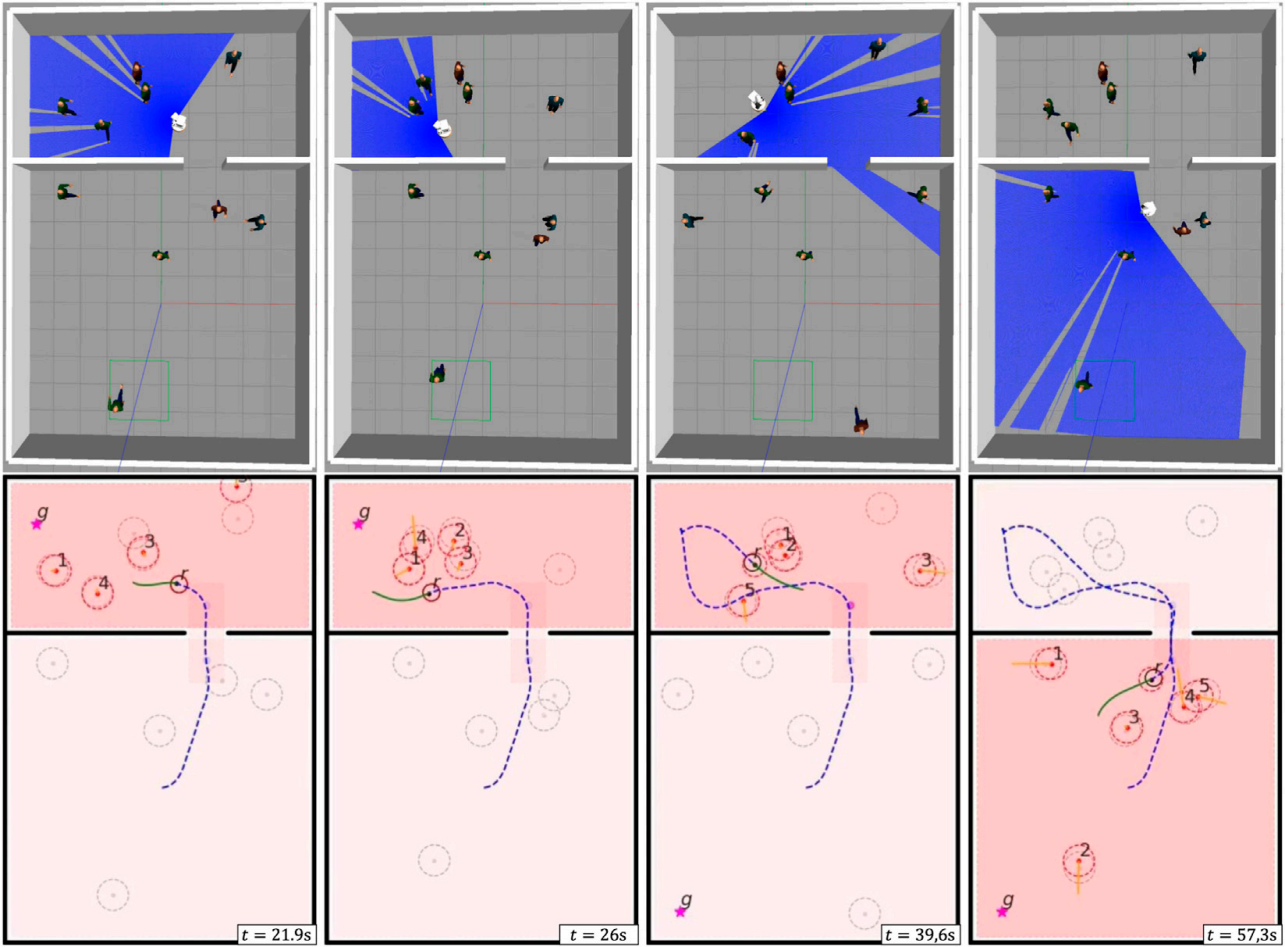
Snapshots of TIAGo navigating a two-room environment with 10 humans. The top row shows the Gazebo simulation, with the laser FOV (blue). The bottom row visualizes the navigation logic: the robot traversed path (dashed blue), planned MPC motion (green), and predicted human trajectories (orange). Labels include the robot (r) , the final goal (g) , and the human tracked by the l -th KF (l) . The robot and the human footprints are shown with dashed red circles, while shaded dashed black circles represent the ground-truth positions of the humans. Convex regions are shown in shaded red, and the region containing the robot is marked with a darker color. Shaded blue dots represent via points, with the target via point in magenta. The sequence demonstrates the robot safely maneuvering through the crowd using motion prediction.

*Simulation 1* (two-room, 10 humans): this scenario demonstrates the baseline performance of the framework in a standard multi-room environment populated by 10 humans. As shown in Figure 10, the robot successfully navigates through convex regions by traversing the computed sequence of via points. The perception system accurately tracks moving humans, allowing the MPC to anticipate their trajectories and execute smooth avoidance maneuvers while progressing toward the final goal. It is worth noting that using DT-CBF-based convex region constraints (see Section 7.1), especially in narrow passages such as doorways, the accumulation of active constraints might slow down progress toward the goal. However, this conservative behavior is a desirable feature as it naturally induces cautious approaches in tight spaces where visibility is inherently limited. In practice, the effect of constraint enforcement on goal attractiveness and overall task completion remains negligible.

**FIGURE 11:**
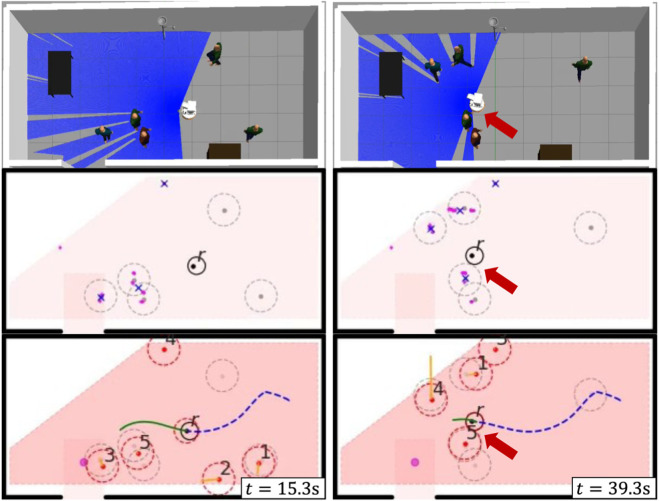
Snapshots of the failure case with laser-only perception. Lacking semantic data, the system incorrectly merges two humans into a single obstacle (left). This results in an unsafe trajectory, causing a false collision detection and emergency stop as the robot approaches and the estimate shifts abruptly (right).

*Simulation 2* (three-room, 15 humans, laser only): this simulation highlights the limitations of relying solely on laser data in a denser crowd of 15 humans. Without semantic information, the laser-based detection fails to distinguish between two humans in close proximity, merging them into a single obstacle cluster. This estimation error leads to an unsafe trajectory plan. As the robot approaches, the laser detects only the closest human, causing an abrupt update in the state estimate that forces an emergency stop (see Figure 11).

**FIGURE 12:**
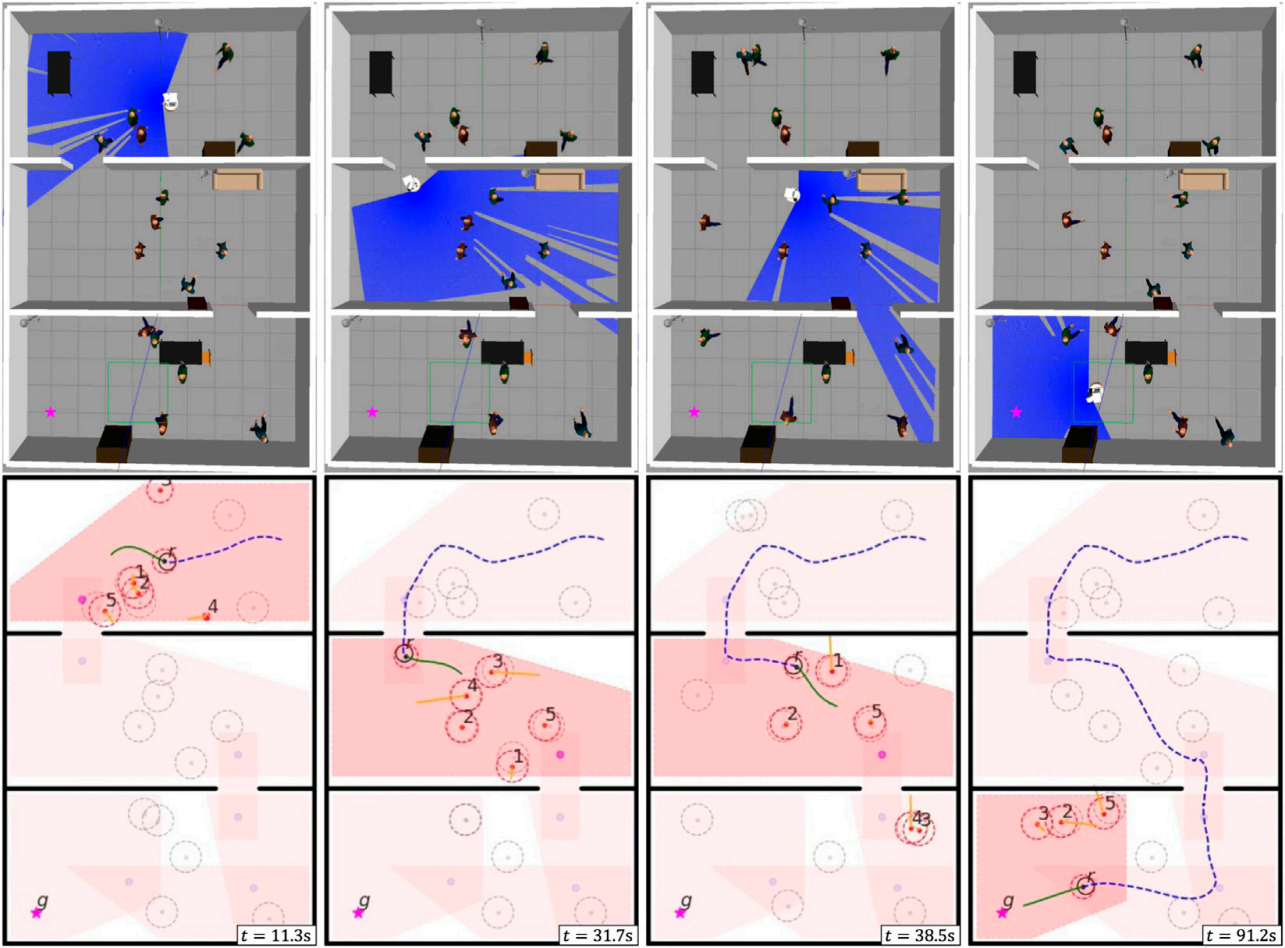
Snapshots of TIAGo navigating a three-room environment with 15 humans. The sequence demonstrates the robot safely maneuvering through the crowd using motion prediction, thereby successfully navigating in a more cluttered environment without collisions.

*Simulation 3* (three-room, 15 humans, laser and camera): to demonstrate the solution to the previous failure, this simulation repeats the exact scenario from Simulation 2 but enables the full sensor fusion pipeline. By integrating RGB-D data, the YOLO-based detector correctly identifies the two individuals, despite their proximity and partial occlusion. This accurate semantic perception allows the crowd estimation module to maintain separate tracks for each human, enabling the MPC to generate a safe and feasible path through the crowd without interruption (see Figure 12).

The aggregated results for all simulations are summarized in Table 1, which reports the success rate of the framework, together with the number and type of failures. A run is considered unsuccessful if a deadlock occurs, preventing the robot from reaching $p^{\text{goal}}$ , or if the estimation module fails. The failure of the estimation module can lead the MPC to enter an unrecoverable infeasibility state (as in Simulation 2) or result in an actual collision with a human. A collision is defined as an overlap between the human footprint and the robot base, specifically when the center-to-center distance is less than $r_{b}+\rho_{h}$ . Given the non-cooperative nature of the crowd, collisions caused by humans walking into a stationary robot are not classified as failures. From the success rate reported in Table 1, it is clear that integrating vision-based detection significantly improves success rates, particularly in dense settings where laser-only perception frequently fails to distinguish grouped individuals. Moreover, failures can be primarily attributed to situations, where humans approach from blind spots as the lack of rear-facing sensors prevents the detection of humans approaching from behind, and to erratic human motion, where unaware pedestrians suddenly change direction when the robot is in close proximity. Deadlocks occur when the MPC becomes trapped in a local minimum typically because the goal is occluded by a pair of static humans or the passage is too narrow to traverse for the robot. In these scenarios, the solver fails to identify a feasible path due to the limited prediction horizon and computational budget. Notably, such deadlocks are less frequent in the laser-only scenario. This is because the laser perception often merges two nearby static humans into a single obstacle cluster; consequently, the system fails due to estimation errors rather than entering a deadlock state.

**TABLE 1:** Performance evaluation comparing laser-only versus combined laser and camera perception across four scenarios varying in the environment layout and crowd density $\left(n_{h}\right)$ . Results are aggregated over 20 runs for each scenario.

$n_{h}$	Rooms	Success rate (%) ↑		Failures ↓ (DL/UI/AC)		Minimum human–robot distance (m) ↑		Travel time (s)	
		Laser	Laser and camera	Laser	Laser and camera	Laser	Laser and camera	Laser	Laser and camera
10	2	65	95	0/3/4	0/0/1	0.95±0.27	0.99±0.22	20.4±5.9	20.7±5.9
	3	55	90	0/3/6	0/1/1	1.05±0.34	0.99±0.3	37.6±10.7	35.6±10.6
15	2	55	85	0/4/5	1/1/1	0.95±0.18	0.88±0.15	21.9±7.4	25.6±11.7
	3	45	70	1/4/6	2/1/3	0.86±0.08	0.89±0.13	34.5±9.8	45.4±16.1

The table reports the success rate (%) , the specific failure causes (DL, deadlock; UI, unrecoverable infeasibility; AC, actual collision), the mean and standard deviation of the minimum human–robot distance, and the travel time, computed across successful runs.

It is important to properly contextualize the safety capabilities of the proposed framework. Our safety claims are mathematically enforced by the DT-CBF constraint formulation, which guarantees that the instantaneous control choices made by the robot are safe with respect to the currently estimated state and predicted human trajectories. However, because human agents can behave unpredictably or even adversarially, maintaining global safety guarantee over an indefinite horizon is fundamentally impossible. Transient feasibility issues and deadlocks will inevitably arise with any local navigation method under these conditions. Thus, the failures observed in our simulations represent edge cases driven by explicitly non-cooperative simulated agents (e.g., humans remaining entirely stationary and blocking narrow passages) and hardware limitations (e.g., rear blind spots), rather than core algorithmic flaws. This distinction is supported by our real-world experiments, when interacting with actual humans who possess natural spatial awareness and do not intentionally navigate into the robot blind spots, the safety enforcement proved highly robust, and no such failures occurred.

Finally, we observed a performance degradation in the most cluttered scenario (three-room, 15 humans). Under these conditions, the system is forced to utilize all K available KFs simultaneously for extended periods. This saturation increases the frequency of rapid changes in the tracked set as humans enter and leave the sensor range, causing repeated re-initialization of the filters and, consequently, less stable motion predictions. Table 1 also reports the minimum human–robot distance and the travel time for each considered scenario, expressed as mean and standard deviation computed across successful runs. As expected, less crowded environments allow the robot to reach the goal more quickly while maintaining a greater distance from nearby humans as more free space is available for navigation. Moreover, since travel time is computed exclusively over successful runs, a lower value does not necessarily reflect better overall performance; rather, it may result from the absence of successes in longer runs, e.g., when more distant goals must be reached or more humans must be avoided. This is clearly illustrated by the most crowded scenarios $\left(n_{h}=15\right)$ in Table 1: in the laser-only perception mode, travel times are lower than in the combined laser and camera mode because the few successful runs contributing to the computation in the laser-only case are the shortest runs, i.e., those with closer goals and/or fewer humans encountered along the path.

### 8.3 Experiments

**FIGURE 13:**
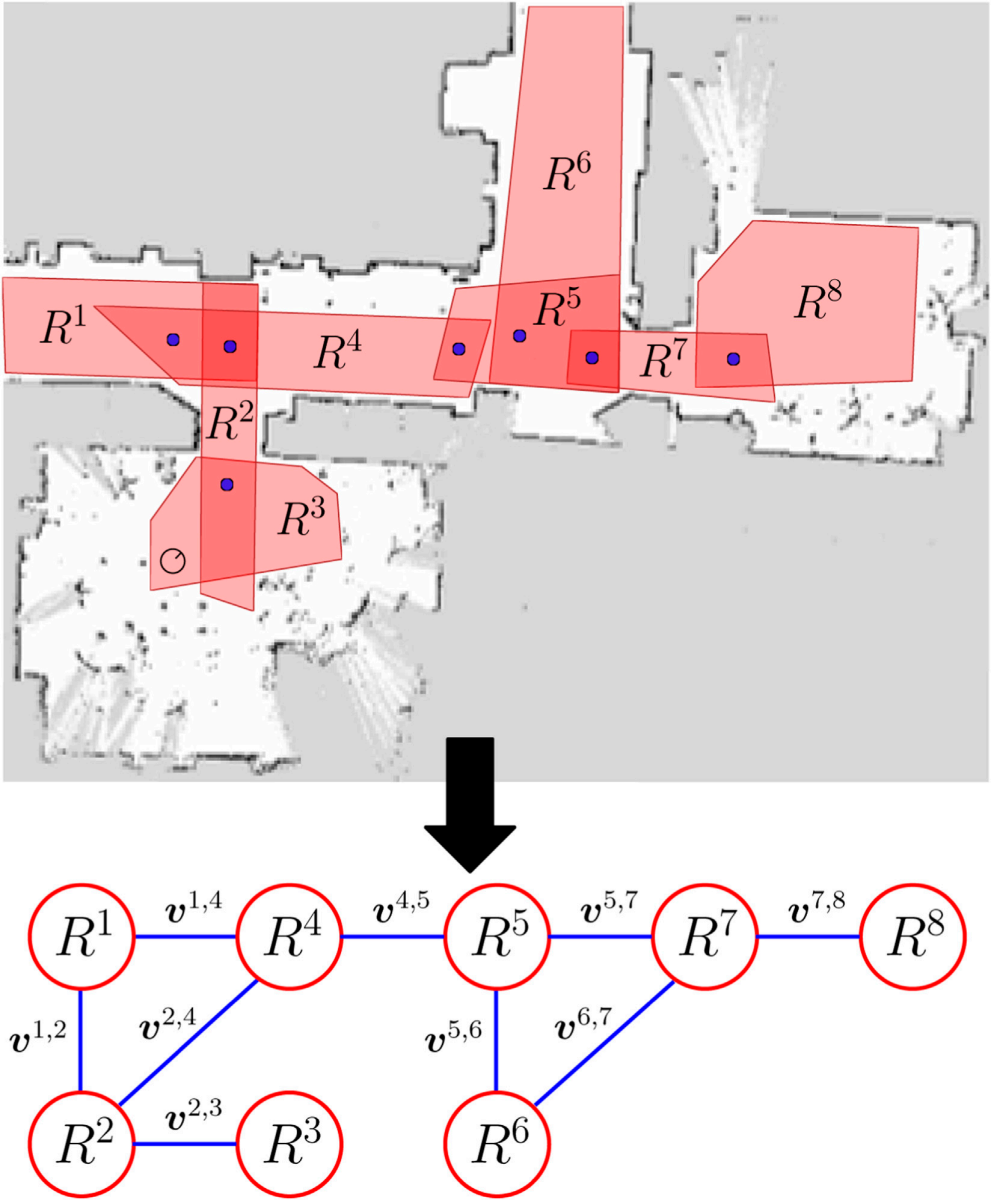
Topological graph generation for global planning starting from the map used in the experiments. (Top) The metric map is decomposed into overlapping convex regions (light red) connected through safe via points (blue dots) located at the intersections. (Bottom) The resulting graph structure G , where regions serve as nodes and via points as edges, enabling the multi-room planner to compute the optimal sequence of areas to traverse.

We further validate the framework through real-world experiments conducted in two distinct environments: a basement setting (depicted in Figure 13) and a departmental hall (featured in the accompanying video).

The environment map is first constructed off-line with the built-in mapping system of TIAGo. It uses laser scanner readings to generate an occupancy grid map, later used for localization. We then cover the free space choosing the convex regions and inserting the via points for each overlapping pair of regions, as illustrated in Figure 13. Although the convex map generation theoretically guarantees that navigable regions are free of static obstacles, real-world environments are inherently dynamic. Objects such as chairs or trash bins may be moved into traversable areas, and structural elements such as door frames can create narrow passages that reduce the robot safety margin. The integration of visual data enables the system to distinguish between humans and unmapped static obstacles. Using the semantic labels provided by the YOLO detector, measurements in $Z_{k}$ are classified as humans when detected using the camera. Measurements without visual confirmation, or with an estimated velocity from the KFs below a predefined threshold, are instead treated as static objects. To prevent overly conservative clearances around static obstacles, we employ a dynamic sizing strategy for the obstacle bounding circle radius $\rho_{h}$ . If an obstacle is classified as a human, this safety margin remains unchanged at 0.35 m. Conversely, when an object is determined to be static, $\rho_{h}$ is gradually reduced to a minimum value of $\rho_{h,\min}=0.1$ m over time, allowing the robot to navigate through tight spaces without being blocked by excessively restrictive constraints.

**FIGURE 14:**
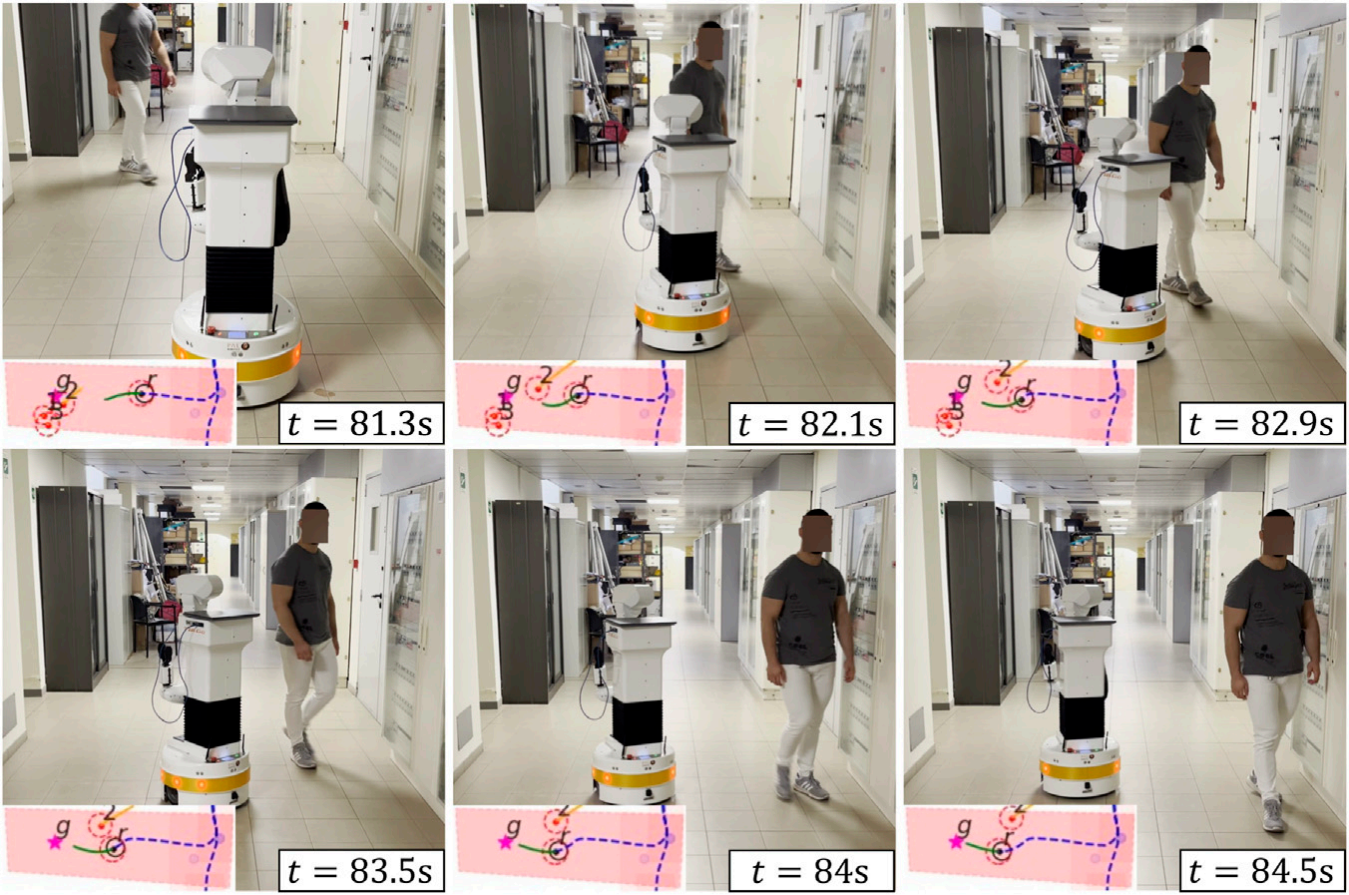
Real-world collision avoidance maneuver with navigation logic in the bottom-left corner. As a human approaches, the robot utilizes the crowd prediction module (orange) to forecast the human trajectory. Consequently, the MPC dynamically re-plans the local motion (green) to deviate from the nominal path, ensuring that safety constraints are met before resuming navigation toward the goal (magenta star).

As expected, the noisiness of the sensors plays a crucial role to correctly estimate and predict the motion of the humans. Nevertheless, the experiments confirm the performance of the proposed approach to perception uncertainties, achieved through appropriate tuning of the DBSCAN parameters used for laser reading clustering and fusion of multi-sensor data. Perception uncertainties and unpredictable agent behavior represent critical challenges in real-world deployments. To navigate these limitations, it is standard practice for fully deployed systems to implement explicit emergency policies and safe backoff mechanisms. Accordingly, our implementation ensures safety by triggering an emergency stop. However, more advanced backoff mechanisms can be readily integrated into the control pipeline. Furthermore, although our physical evaluation is naturally constrained by the standard sensor suite equipped on the TIAGo platform, this setup represents typical (and economically reasonable) equipment for mobile robots. Due to the modular structure of our perception pipeline, incorporating measurements from additional sensors to expand data fusion comparisons, such as secondary RGB-D cameras to eliminate rear blind spots or high-resolution depth sensors for richer volumetric mapping, is a straightforward extension. A remarkable collision avoidance maneuver executed by our framework during experiments is depicted by the sequence of snapshots in Figure 14.

**FIGURE 15:**
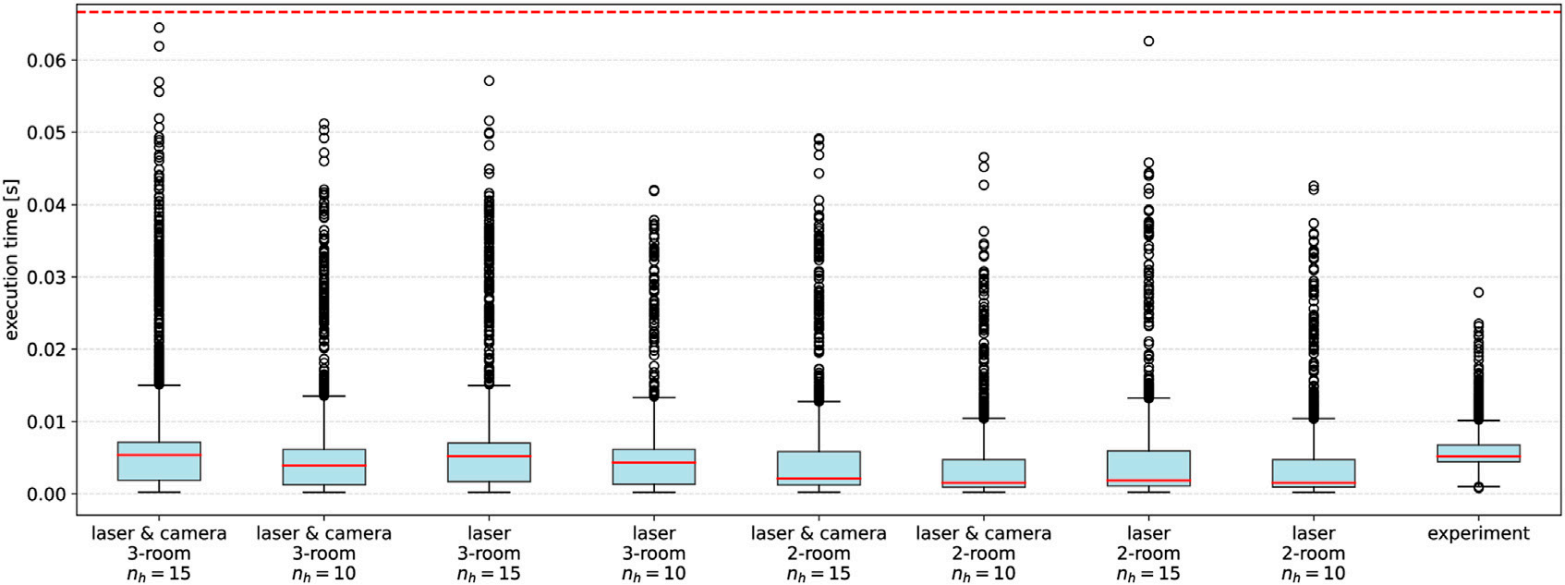
Execution times of the motion generation module considering all the runs over eight simulated scenarios and the experiment. The dashed red line represents the maximum available time per iteration of 0.067 s, allowing the motion generation module to compute commands at a 15 Hz frequency.

Finally, Figure 15 confirms the computational efficiency of the proposed motion generation module. For each of the eight simulated scenarios, execution times are aggregated across all available runs; for the real-world validation, they are measured over the experiment partially shown in Figure 14 (the complete experiment is available in the accompanying video). Notably, all iterations are computed within the allotted time budget of 0.067 s (at least 15 Hz), and, aside from occasional outliers, the motion generation time remains below this threshold.

## 9 Conclusion

In this study, we presented a sensor-based MPC scheme for safe multi-room crowd navigation. Information from both 2D laser and RGB-D camera is processed, and KFs are employed to estimate the state of surrounding humans and infer their future motion. Contextually, given the current robot position and a goal position in a multi-room environment, a planner finds the sequence of rooms to reach the desired position which is given to an MPC that safely drives the robot using DT-CBF-based collision avoidance constraints. We demonstrated via simulations and experiments that introducing vision-based information allows the robot to overcome some deadlock conditions and leads to better performance. Possible future work may concern the following: (i) the introduction of different human motion prediction models in the MPC, e.g., a unicycle model, to better predict the intentions of humans and other possible dynamic agents such as robots; (ii) the further improvement in the perception module by introducing non-human object detection and reconstruction; (iii) the development of social and context-aware behavior, such as giving way or getting in line; (iv) the extension to a dynamic graph construction to allow the online topological planner to re-plan in case of environmental changes, e.g., find a new route to the goal if a closed door is encountered; (v) the integration of non-vision-based sensors, such as radar or acoustic microphone arrays, to further enhance human detection robustness against severe occlusions and sensor blind spots.

## Data Availability

The raw data supporting the conclusions of this article will be made available by the authors, without undue reservation.

## Footnotes

1 The current formulation is intended to solve a navigation task limited to reach a target planar location (i.e., position convergence); therefore, we do not explicitly regulate terminal orientation. However, extending the framework to support general pose-to-pose navigation is straightforward. It requires modifying the task error definition $e_{i|k}$ to include the heading error $\left|\theta_{\text{des}}-\theta \right|$ and appropriately expanding the dimension of the weighting matrices Q and $Q_{N}$ .
