# Supplementary material for: Crowd navigation in a multi-room environment: a model predictive control framework for mobile robots
Source: Front Robot AI. 2026 Jul 27;13:1812386. doi: 10.3389/frobt.2026.1812386 (PMC13453804; doi:10.3389/frobt.2026.1812386)
Supplement: Supplementary file 1 [file Supplementaryfile1.pdf]

Appendix

We provide here pseudocode descriptions of the following functionalities: the computation of the sequence of convex regions (Algorithm 1), the extraction of measurements from laser (Algorithm 2) and camera (Algorithm 3) sensors, and the motion generation procedure (Algorithm 4).

Algorithm 1 computeSequence

Input: $\mathcal{G}$, $\mathcal{R}$, $\boldsymbol{p}^{\text{goal}}$, $\boldsymbol{p}$, $\mathcal{A}$

Output: $\mathcal{S}$

$R^c \leftarrow$ getRegion$(\mathcal{R}, \boldsymbol{p})$
if isInside$(\boldsymbol{p}^{\text{goal}}, \mathcal{A})$ **then**
    $R^{\text{goal}} \leftarrow$ getRegion$(\mathcal{R}, \boldsymbol{p}^{\text{goal}})$
else
    $R^{\text{goal}} \leftarrow$ getClosestRegion$(\mathcal{R}, \boldsymbol{p}^{\text{goal}})$
end if
$\mathcal{S} \leftarrow$ Dijkstra$(\boldsymbol{p}, R^c, R^{\text{goal}}, \mathcal{G})$

Algorithm 2 laserDetection

Input: $\mathcal{O}_k^{\text{las}}$, $\mathcal{A}$

Output: $\mathcal{Z}_k^{\text{las}}$

for $\boldsymbol{o}^{\text{las}} \in \mathcal{O}_k^{\text{las}}$ **do**
    $\boldsymbol{p}^l \leftarrow$ extractPosition$(\boldsymbol{o}^{\text{las}})$
    if isInside$(\boldsymbol{p}^l, \mathcal{A})$ **then**
        $\mathcal{L}_k \leftarrow$ addMeasure$(\boldsymbol{p}^l)$
    end if
end for
$\mathcal{D}_k \leftarrow$ DBSCAN$(\mathcal{L}_k)$
$\mathcal{Z}_k^{\text{las}} \leftarrow$ extractRepresentativePoints$(\mathcal{D}_k)$

Algorithm 3 visionDetection

Input: $\mathcal{O}_k^{\text{cam}}$, $\mathcal{A}$, $\boldsymbol{R}_k^c$, $\boldsymbol{p}_k^c$, $\boldsymbol{K}$
Output: $\mathcal{Z}_k^{\text{cam}}$

$\mathcal{B}_k \leftarrow \text{extractBoundingBoxes}(\mathcal{O}_k^{\text{cam}})$
for $\boldsymbol{b}' \in \mathcal{B}_k$ **do**
  $\boldsymbol{c}' \leftarrow \text{getCenter}(\boldsymbol{b}')$
  $d' \leftarrow \text{getDepth}(\boldsymbol{b}', \mathcal{O}_k^{\text{cam}})$
  $\boldsymbol{p}^l \leftarrow \text{reconstructPosition}(\boldsymbol{c}', d', \boldsymbol{R}_k^c, \boldsymbol{p}_k^c, \boldsymbol{K})$
  if $\text{isInside}(\boldsymbol{p}^l, \mathcal{A})$ **then**
    $\mathcal{Z}_k^{\text{cam}} \leftarrow \text{addMeasure}(\boldsymbol{p}^l)$
  end if
end for

Algorithm 4 motionGeneration

Input: $\mathcal{S}$, $\boldsymbol{p}^{\text{goal}}$, $\bar{e}$

$R^{\text{curr}} \leftarrow \text{getCurrentRegion}()$
while $\mathcal{S}$ is not empty **do**
  $R^\ell \leftarrow \text{getNextRegion}(\mathcal{S})$
  $\boldsymbol{p}^{\text{des}} \leftarrow \text{getTargetViaPoint}(R^\ell)$
  $\boldsymbol{x} \leftarrow \text{getState}()$
  $\mathcal{P} \leftarrow \text{getHumansPrediction}()$
  while $\text{isOutside}(\boldsymbol{x}, R^\ell)$ **do**
    $\boldsymbol{u} \leftarrow$Model Predictive Control (MPC)$(\boldsymbol{x}, \boldsymbol{p}^{\text{des}}, \mathcal{P}, R^{\text{curr}})$
    $\text{sendRobotCommands}(\boldsymbol{u})$
    $\boldsymbol{x} \leftarrow \text{getState}()$
    $\mathcal{P} \leftarrow \text{getHumansPrediction}()$
  end while
  $R^{\text{curr}} \leftarrow R^\ell$
end while
$\boldsymbol{p}^{\text{des}} \leftarrow \boldsymbol{p}^{\text{goal}}$
while $\text{taskError}(\boldsymbol{p}^{\text{des}}, \boldsymbol{x}) \geq \bar{e}$ **do**
  $\boldsymbol{u} \leftarrow \text{MPC}(\boldsymbol{x}, \boldsymbol{p}^{\text{des}}, \mathcal{P}, R^{\text{curr}})$
  $\text{setCommands}(\boldsymbol{u})$
  $\boldsymbol{x} \leftarrow \text{getState}()$
  $\mathcal{P} \leftarrow \text{getHumansPrediction}()$
end while
